# Improved phosphorus MRSI acquisition through compressed sensing acceleration combined with low-rank reconstruction

**DOI:** 10.1007/s10334-024-01218-y

**Published:** 2024-12-27

**Authors:** Julien Songeon, François Lazeyras, Thomas Agius, Oscar Dabrowski, Raphael Ruttimann, Christian Toso, Alban Longchamp, Antoine Klauser, Sebastien Courvoisier

**Affiliations:** 1 Department of Radiology and Medical Informatics, Faculty of Medicine, University of Geneva, Geneva, Switzerland; 2 CIBM Center for Biomedical Imaging, University Hospital of Geneva, Bd de la Tour 8, 1205 Geneva, Switzerland; 3 Department of Vascular Surgery, Lausanne University Hospital and University of Lausanne, Lausanne, Switzerland; 4 Visceral and Transplant Surgery, Department of Surgery, Geneva University Hospitals and Medical School, Geneva, Switzerland

**Keywords:** Acceleration, Compressed sensing, Low rank, Regularization, Phosphorus magnetic resonance spectroscopic imaging (^31^P-MRSI)

## Abstract

**Objectives:**

Phosphorus-31 magnetic resonance spectroscopic imaging (^31^P-MRSI) is a non-invasive tool for assessing cellular high-energy metabolism in-vivo. However, its acquisition suffers from a low sensitivity, which necessitates large voxel sizes or multiple averages to achieve an acceptable signal-to-noise ratio (SNR), resulting in long scan times.

**Materials and methods:**

To overcome these limitations, we propose an acquisition and reconstruction scheme for FID-MRSI sequences. Specifically, we employed Compressed Sensing (CS) and Low-Rank (LR) with Total Generalized Variation (TGV) regularization in a combined CS–LR framework. Additionally, we used a novel approach to k-space undersampling that utilizes distinct pseudo-random patterns for each average. To evaluate the proposed method’s performance, we performed a retrospective analysis on healthy volunteers’ brains and *ex-vivo* perfused kidneys.

**Results:**

The presented method effectively improves the SNR two-to-threefold while preserving spectral and spatial quality even with threefold acceleration. We were able to recover signal attenuation of anatomical information, and the SNR improvement was obtained while maintaining the metabolites peaks linewidth.

**Conclusions:**

We presented a novel combined CS–LR acceleration and reconstruction method for FID-MRSI sequences, utilizing a unique approach to k-space undersampling. Our proposed method has demonstrated promising results in enhancing the SNR making it applicable for reducing acquisition time.

**Supplementary Information:**

The online version contains supplementary material available at 10.1007/s10334-024-01218-y.

## Introduction

Phosphorus-31 magnetic resonance spectroscopy (^31^P-MRS) is a non-invasive technique that provides valuable information on cellular high-energy metabolism in-vivo [1–5]. When combined with spatial phase encoding, ^31^P-MRS imaging (MRSI) allows for multi-voxel acquisition, enabling metabolite mapping across the entire field-of-view (FoV) [6]. In addition, ^31^P-MRS can provide an estimation of intracellular pH (pHi) from the chemical shifts, which can be utilized for pH mapping [7–11]. The unique information obtained through phosphorus spectroscopy has generated increasing interest in metabolite mapping in-vivo. These features find widespread applications in studying various medical conditions, such as diabetes [12–14], Alzheimer’s disease [15, 16], migraine [17, 18], oxidative stress [19], muscular dystrophies [20–22], and cancer [23–25].

Despite the advantages of ^31^P-MRS, its acquisition displays a lower relative sensitivity than hydrogen-1 (^1^H) at a constant magnetic field, necessitating larger voxel sizes to achieve a sufficient signal-to-noise ratio (SNR) while maintaining an acceptable scan time for the patient [26, 27]. Furthermore, to compensate for a low SNR, multiple averages can be employed, but this comes at the expense of prolonging the scan time. Lengthy acquisition could be overcome using acceleration techniques, such as Compressed Sensing (CS), which has been widely studied in ^1^H-MRSI and is investigated in other nuclei as well [28].

Compressed Sensing (CS) is an acquisition acceleration method involving random k-space undersampling [29, 30]. This technique has been demonstrated to significantly reduce scan time while maintaining high image quality, making it attractive in clinical settings [31]. The application of CS in ^1^H imaging has been extensively investigated and has been shown to be effective for a variety of applications [32–35]. In parallel, CS has been applied in spectroscopy, specifically in 2D and 3D ^1^H-MRSI [36–38], enabling fast high-resolution metabolic mapping of the brain [39–41]. The success of CS in ^1^H imaging and spectroscopy has opened up opportunities to explore its application to other nuclei, such as ^13^C to accelerate hyperpolarized 3D-MRSI acquisition that requires short acquisition time [42–45].

The Low-Rank (LR) method has been employed to reconstruct accelerated CS data. This approach is particularly advantageous for application requiring an improved SNR, as they can effectively denoise as well [46, 47]. LR method described as union-of-subspaces was successfully applied to ^1^H-MRSI [48]. Likewise, LR tensor model has allowed sparse ^31^P-MRSI acquisition scheme to be used, resulting in faster acquisition with high resolution and improved SNR [49]. The combination of CS–LR has been increasingly applied to MRI [50–52] and ^1^H-MRSI [39–41], benefiting scan time acceleration while improving image quality and quantitation accuracy. By combining the CS–LR method with total generalized variation (TGV) for data regularization, the acquisition time of MRSI data can be significantly reduced without compromising the quality of the reconstructed image [53, 54].

The current study aims to demonstrate the feasibility of this novel approach that combines compressed sensing and low-rank techniques with total generalized variation regularization to ^31^P-MRSI data obtained via 3D FID-MRSI sequence. Thus, the focus is on situations where the signal-to-noise ratio (SNR) is low, particularly for molecules such as ATP, which serve as a marker of cellular viability assessment before transplantation [55]. The proposed approach utilizes elliptical encoding and weighted averages, combined to an independent pseudo-random undersampling scheme for each average. The resulting FID measurement is then incorporated into the CS–LR reconstruction. Following quantification of spatio-spectral reconstruction using a state-of-the-art low-SNR-able method [56], we evaluated the spatial information preservation relatively to the acceleration. The CS–LR approach was applied under two distinct conditions: first, in-vivo on ten human brains, and second, *ex-vivo* on two perfused kidneys from a single pig, to demonstrate its versatility.

## Materials and methods

### Acceleration and reconstruction

#### Undersampling with weighted averaging

**Fig. 1:**
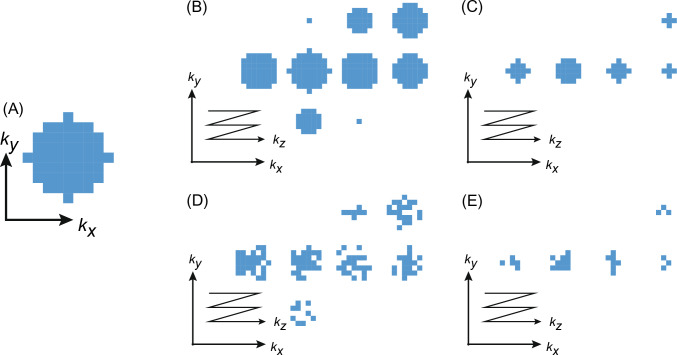
Example of k-space filling of a 10 × 10 × 10 voxel acquisition, illustrating the differences between fully sampled k-space and randomly undersampled k-space. Panel **A** shows k-space center *k*_*z*_ = 0 plane of fully sampled k-space, while panels **B** and **C** depict the first and the 10th (out of 24) weighted average, respectively. The impact of undersampling can be seen in panels (**D**) and (**E**), which display unique randomly undersampled k-space for an acceleration factor of 2

Initially, ^31^P-MRSI data were acquired with a weighted average elliptical sampled k-space [57], as illustrated in Fig. 1A, B and C. This fully sampled k-space was a posteriori undersampled. To achieve this, the Fourier domain was characterized by a radius *q*, which was expressed as *q* = $$\sqrt {\left( {{\raise0.7ex\hbox{${kx}$} \!\mathord{\left/ {\vphantom {{kx} {kx\max}}}\right.\kern-0pt} \!\lower0.7ex\hbox{${kx_{\max} }$}}} \right)2 + \left( {{\raise0.7ex\hbox{${ky}$} \!\mathord{\left/ {\vphantom {{ky} {ky}}}\right.\kern-0pt} \!\lower0.7ex\hbox{${ky}$}}_{\max} } \right)2 + \left( {{\raise0.7ex\hbox{${kz}$} \!\mathord{\left/ {\vphantom {{kz} {kx\max }}}\right.\kern-0pt} \!\lower0.7ex\hbox{${kz_{\max }}$}}} \right)} 2$$. The pseudo-random sampling was constructed, such that the density distribution had a density of *q*^−1^. To simulate the accelerated acquisition, we removed retrospectively **k**_*i*_ values of the 3D phase-encoded measurements, while maintaining a 20% fully sampled center of the k-space for $$q \le \frac{1}{5}$$ [39].

The acceleration is quantified using the acceleration factor (AF), which represents the inverse of the undersampling factor. The pseudo-random undersampling was performed for each weighted average individually. Figure 1 depicts the fully sampled k-space for two averages [centered (B) and 10th (C) averages] along with potential undersampling patterns (D) and (E), respectively. Employing different pseudo-random undersampling patterns for each average allow for a reciprocal distribution of acceleration across the averages, whereby each average being undersampled with the same factor. Conversely, applying a fixed undersampling pattern to each weighted average would lead to a k-space position being suppressed n-times if it is close to the center, while a point close to the periphery would only be suppressed once. Using a fixed undersampling pattern would necessitate an iterative calculation of acceleration after each suppression. Furthermore, utilizing different pseudo-random undersampling patterns for each average ensures that the remaining points are sparse enough to meet the requirements of compressed sensing [30, 58].

#### CS–LR reconstruction

The CS–LR method presented in this article has been developed based on the work of Klauser et al. [39–41]. In MRSI, the signal measured by a coil element at a specific time *t* and Fourier-space position **k** can be expressed using the integral equation shown in Eq. (1)

1 $$s\left( {{\varvec{k}},t} \right) = \int\limits_{{\Omega \subset {\mathbb{R}}^{3} }} {\rho \left( {{\varvec{r}},t} \right)C\left( {\varvec{r}} \right)e^{{ - 2\pi it\Delta B0\left( {\varvec{r}} \right)}} e^{{ - 2\pi i{\varvec{kr}}}} {\text{d}}{\varvec{r}}} .$$

Here, the local transverse magnetization *ρ*(**r***,t*) is modulated by the coil sensitivity profile *C*(**r**) and is affected by the map of field inhomogeneity in Hz ∆*B*_0_(**r**) over the measured object Ω. In the present experiment, we employed a single coil element; therefore, *ρ*(**r***,t*) and *C*(**r**) could be combined. Although the sensitivity profile of a volumetric coil is uniform, this cannot be assumed for a loop coil. This implies a weighting of the transverse magnetization with the coil sensitivity profile. For a weighted average *a*, Eq. (1) can be discretized to give the relationship between the measured signal **s**_*a*_, the Fourier transform operator $\mathcal{F}a$ which includes the Fourier transform as well as the unique undersampling pattern of each average, the frequency shift operator created by the inhomogeneity $\mathcal{B}$, and the local transverse magnetization **ρ**

2 $${\mathbf{s}}_{a} = FaB\rho + \in .$$

*ϵ* accounts for the noise and is assumed to be Gaussian. The CS–LR reconstruction provides a solution to the inverse problem that is the reconstruction of the local transverse magnetization **ρ** knowing $\mathcal{F}$_*a*_$\mathcal{B}$ with **s**. The method assumes that the MRSI data can be represented by a low-rank matrix, which decomposes the spectral data into a small number of dominant components *K* [39–41, 59]. The low-rank reconstruction is presented in the following equations:

3 $$\rho_{{{\varvec{l}},{\varvec{j}}}} = \mathop \sum \limits_{n = 1}^{K} U_{l,n} V_{n,j }$$

4 $$\rho = {\varvec{UV}}\user2{.}$$

*ρ*_*l,j*_ can be represented as a linear combination of a small number of characteristic spectra that are spatially distributed across the measured volume. *T* represents the time series, with *j* = 1*,…,T*; and *N*^*r*^ represents the number of **r** vectors in the image space with *l* = 1*,…,N*^*r*^. The matrix **V** ∈ $C$ ^*K*×*T*^ contains the finite set of characteristic time series, and **U** ∈ $C$ ^*Nr* ×*K*^ represents their spatial distribution. The proposed method is well suited for processing MRSI data due to the finite number of metabolite resonances that are assumed to be partially separable of their spatial distribution [46, 49]. Moreover, noise within the MRSI dataset is typically stochastic and lacks specific spatial distributions, which renders the fitting of a low-rank model to be an effective denoising tool. Regularization applied on spatial metabolite components permits the denoising in space while preserving edges and enforcing data consistency [58]. The inverse problem is written as

5 $$\mathop {\arg \min }\limits_{{{\varvec{U}},{\varvec{V}}}} \mathop \sum \limits_{a} {\varvec{s}}a - \mathcal{F}a\mathcal{B}{\varvec{UV}}_{2}^{2} + \lambda \mathop \sum \limits_{n = 1}^{K} TGV^{2} {\varvec{U}}\user2{.}$$

The spatial and temporal components are obtained by minimizing the inverse problem in conjunction with total generalized variation (TGV) spatial regularization method [53]. In our research, the values of *K* = 5 and *λ* = 0*.*001 were determined empirically yielding optimal outcome. The CS–LR reconstruction with and without acceleration will be compared to traditional FFT transformation. The reconstruction was performed in Matlab (The MathWorks, Natick, Massachusetts, USA), and required 5 h computation time for the 3D-MRSI using 8 cores of a 3.00 GHz Intel(R) Xeon(R)-E5 CPU, and 124 GB of RAM.

### MRI measurements

In-vivo measurements were conducted on a clinical Prisma-fit 3 T MRI scanner (Siemens Healthineers, Erlangen, Germany) that is equipped with multinuclear capabilities. Notably, no proton decoupling was applied.

#### Brain

The results were obtained retrospectively from anonymous data, with informed consent from all ten volunteers. The study was approved by the ethics committee and followed the Declaration of Helsinki and local regulations (swissethics, CCER). [56]. T1-weighted MP-RAGE acquisition was employed to obtain anatomical reference ^1^H images. A dual-frequency (^1^H and ^31^P) birdcage head-coil with quadrature transceive geometry (Clinical MR solutions, Brookfield, WI) was used for scanning. A 10 × 10 × 10 matrix was employed for the acquisition of 3D ^31^P-MRSI of the whole brain, with an isotropic field-of-view (FoV) dimension of 250 mm yielding a nominal spatial isotropic resolution of 25 mm. The sequence consisted of a rectangular excitation pulse of 0.25 ms with a flip angle of 45^°^ (calculated on the PCr maximum amplitude), a repetition time (TR) of 1500 ms, and an echo time (TE) of 0.5 ms with 24 weighted averages [55]. The receive bandwidth was 4000 Hz for 2048 sampling points, and the fully sampled elliptical MRSI acquisition with weighted averages lasted 37 min.

#### Kidney

 MRSI was performed on *ex-vivo* pig kidneys from one subject as part of a study evaluating the viability of marginal grafts. All procedures adhered to the Swiss animal care and use guidelines to ensure ethical treatment and welfare. To enable imaging, the organs were perfused using a homemade MR-compatible perfusion system [55, 60], featuring a single linearly polarized RF squared loop (measuring 5.2 inches or 13.2 cm on each side), securely fixed to the bottom of the perfusion tank. The coil was interfaced with a specially designed transceiver that allowed for both ^1^H imaging and ^31^P spectroscopy (Clinical MR Solutions, Brookfield, WI). The body coil was used to perform ^1^H imaging with T2-weighted sequence (turbo SE, TR 6530 ms, TE 110 ms, 2 mm slices) for kidney localization and structural imaging. The 3D ^31^P-MRSI was acquired with a FoV of 250 mm × 250 mm × 160 mm and a matrix size of 16 × 16 × 8, yielding a nominal spatial resolution of 15.6 mm × 15.6 mm × 20 mm. The TR was set to 1000 ms, the flip angle was 45° (calculated on the Pi maximum amplitude), the echo delay was 0.6 ms, and the receiver bandwidth was 4000 Hz for 2048 sampling points. The acquisition employed elliptical encoding with 18 weighted averages, and the acquisition time was 45 min. The chemical shift signal was referenced to the inorganic phosphate (Pi) resonance at 5.2 ppm, which can be considered homogeneously distributed over the surface of the coil.

### Spectral quantification and metabolites mapping

^31^P-MRSI spectra reconstructed by CS–LR were analyzed and quantified individually using the openly available ^31^P-SPAWNN method [56]. The method uses convolutional neural networks (CNN) to estimate spectral parameters (phase, linewidth, frequency, etc.) and metabolite concentrations from each voxel/spectra. This quantification could be used to simulate back the source spectra using the physical model to evaluate the quality of quantification. Quantifications allow to generate metabolites concentration images (real and positive values) or ratio of concentration images of the source sample. The quantified metabolites included phosphocreatine (PCr), inorganic phosphate (Pi), and adenosine triphosphate (*α*-ATP, *β-*ATP, and *γ*-ATP). Additionally, the phosphomonoesters (PME) consisting of phosphocholine (PC) and phosphoethanolamine (PE), and the phosphodiesters (PDE) consisting of glycerophosphocholine (GPC) and glycerophosphoethanolamine (GPE), as well as nicotinamide adenine dinucleotide (NAD + and NADH) and membrane phospholipids (Mp) were also included in the quantification. MRSI data did not have an internal reference for quantification, and therefore, the results will only be presented as a ratio of metabolite concentrations.

### Evaluation of performance

#### Error quantification

The performance of compressed sensing with the CS–LR model was evaluated using the normalized root-mean-square error (NRMSE). NRMSE was calculated to assess the accuracy of the ratio estimation obtained with the CS–LR method with acceleration compared to the CS–LR method without acceleration (fully sampled). The computation was performed with the acceleration factor of 1.11, 1.25, 1.42, 1.66, 2., 2.5, 2.94, and 3.; corresponding to 90%, 80%, 70%, 60%, 50%, 40%, 34%, and 33% of the fully sampled k-space, respectively

6 $${\text{NRMSE}} = \sqrt {\frac{{\mathop \sum \nolimits_{i} \left| {m_{i}^{no\;acc.} - m_{i}^{acc.} } \right|^{2} }}{{\mathop \sum \nolimits_{i} m_{i}^{no\;acc.} }}} .$$

The sum over the index *i* was taken for all the voxels in the brain or the kidney for each metabolite *m*. For the brain, the ratio of metabolite concentration was calculated with respect to PCr across the whole brain for the ten subjects. For the kidney, the ratio was computed with respect to Pi.

#### Spectral evaluation

The present study evaluates the spectral quality by calculating the SNR for the voxels of interest, both for the original data and for the CS–LR methods implemented with and without acceleration. Specifically, the SNR is computed for the brain data by utilizing the PCr peak intensity as the reference signal and divided by the noise standard deviation. We also evaluated the SNR gain of the CS–LR method relatively to the FFT of the original data: $SNR\left( {PCr_{CS - LR} } \right)/SNR\left( {PCr_{FFT} } \right)$. For comparison, we also compared the SNR gain of the apodized spectrum using a matched Gaussian filter based on the full width half maximum of the PCr peak: $SNR\left( {PCr_{FFTapod} } \right)/SNR\left( {PCr_{FFT} } \right)$. For the kidney data, the PME peak is employed for this purpose. Additionally, linewidth of all resonances was measured to determine whether the CS–LR method causes peak broadening.

#### Spatial evaluation

The study assesses the spatial quality of the metabolic mapping quantified on the reconstruction done with either the FFT of the fully sampled data or with the CS–LR using various acceleration factors. The sharpness of the image was evaluated by computing the image gradient. To determine the sharpness and complexity of the image, the Sobel method presented by Yu et al. [61] was employed. Given that the CS–LR scheme addresses the inverse problem and incorporates spatial regularization for finding a solution in the spatial-spectral domain, we evaluated the accuracy and preservation of spatial information on the metabolite quantification maps (real and positive values). This was done by computing the image gradient in the three axes on the metabolite quantification maps following full reconstruction and subsequent spectral quantification. Subsequently, the root-mean-square (RMS) and its standard deviation of the edge magnitudes were computed from the spatial information values for each metabolite and method. Edge magnitudes were compared across acceleration factors of CS–LR using analysis of variance (ANOVA). Post hoc contrasts were then used to identify significant differences between steps of increasing acceleration factor and the FFT of the original data, using Dunn–Šidák correction to control the family-wise error rate.

## Results

**Fig. 2:**
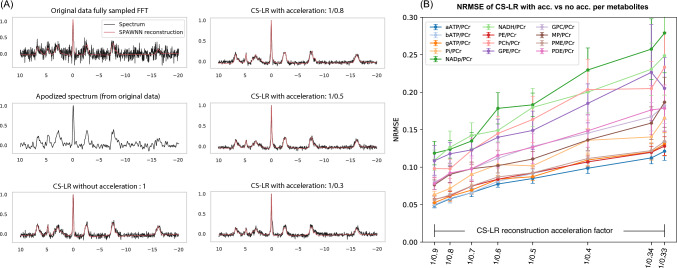
Brain ^31^P-MRSI spectra: effect of reconstruction and acceleration. **A** Spectra from a brain voxel: original FFT, matched filter (apodized), and CS–LR reconstructions at acceleration factors 1.25, 2, and 3. **B** Normalized root-mean-square error (NRMSE) of metabolite concentration ratios with acceleration, benchmarked against the non-accelerated method

Figure 2 presents the brain results obtained from the ten healthy volunteers. Figure 2A illustrates typical reconstructed spectra used in the subsequent analysis. The example is arbitrarily selected from a single voxel located in the frontal region of subject 8 (see slice in Fig. 4A) and compares different methodologies qualitatively. The comparison includes the original spectrum obtained via Fourier transform, its apodized version using a Gaussian matched filter, as well as spectra generated using the CS–LR method without acceleration and with acceleration factors of 1.25, 2.0, and 3.0. Figure 2B presents the normalized mean square error (NRMSE) obtained with the CS–LR method with acceleration compared to the CS–LR method without acceleration. The reduction in k-space sampling is associated with a linear decrease in scan time, resulting in acquisition of approximately 33, 29, 26, 22, 19, 15, and 12 min, respectively. The NRMSE was computed on all the voxels; data points correspond to the mean and SD values across the ten subjects. The NRMSE exhibited a monotonic trend, closely resembling a linear relationship with the acceleration factor. In the case of ATP metabolites, the error rate was approximately 5% at an acceleration of 1.11, increasing to 10% at an acceleration of 3. The concentration estimation of Pi, PME, and PDE presented an initial error rate between 7 and 10%, which rose to a range of 13% to 17%.

**Fig. 3:**
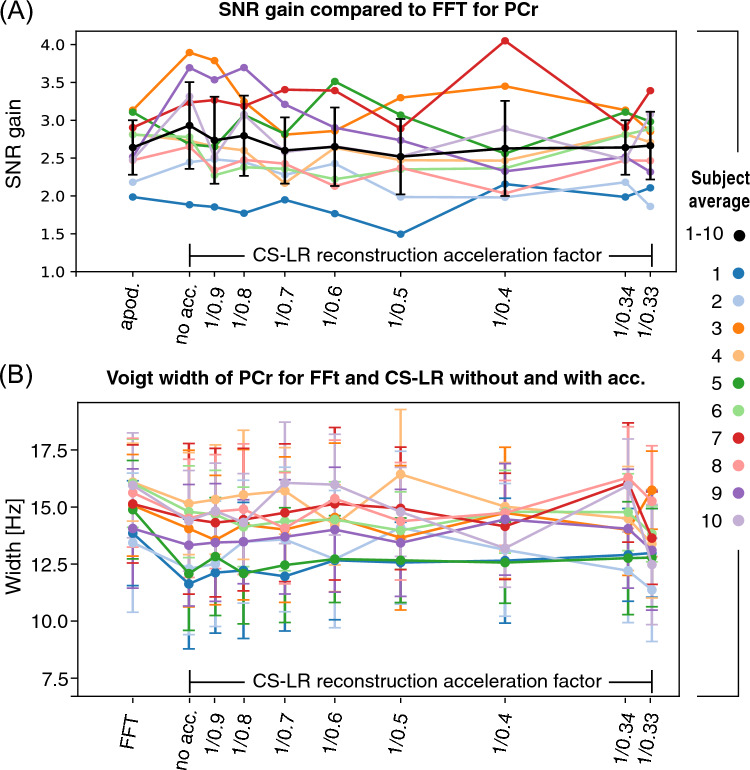
Outcome summary with ten participants. **A** Signal-to-noise ratio (SNR) relative to phosphocreatine (PCr); **B** peak width of PCr for the FFT of the original data, the CS–LR method without and with various acceleration factors

Figure 3 illustrates spectral metrics relative to phosphocreatine (PCr) for all subjects or brains, comparing both the Fourier transform of the original measurement and the CS–LR method across increasing acceleration factors. All three plots share the same legend. Figure 3A illustrates the SNR gain relative to the original FFT reconstruction for spectra enhanced by matched filter apodization or reconstructed using the CS–LR method. Applying the CS–LR approach resulted in a two-to-threefold increase in SNR, consistent across all acceleration factors. This SNR improvement is comparable to that achieved with a matched apodization filter. Finally, Fig. 3B displays the linewidth values of PCr for all ten volunteers for original data FFT and CS–LR method. Similar linewidth results for Pi, ATP, and PME are shown in supplementary material Fig. S2.1.

The CS–LR method led to a significant improvement in SNR similar to a matched filter method, uniformly across all participants. Importantly, no broadening of metabolite linewidth was detected for CS–LR compared to the original FID data, as indicated by consistent linewidth values across all acceleration factors.

**Fig. 4:**
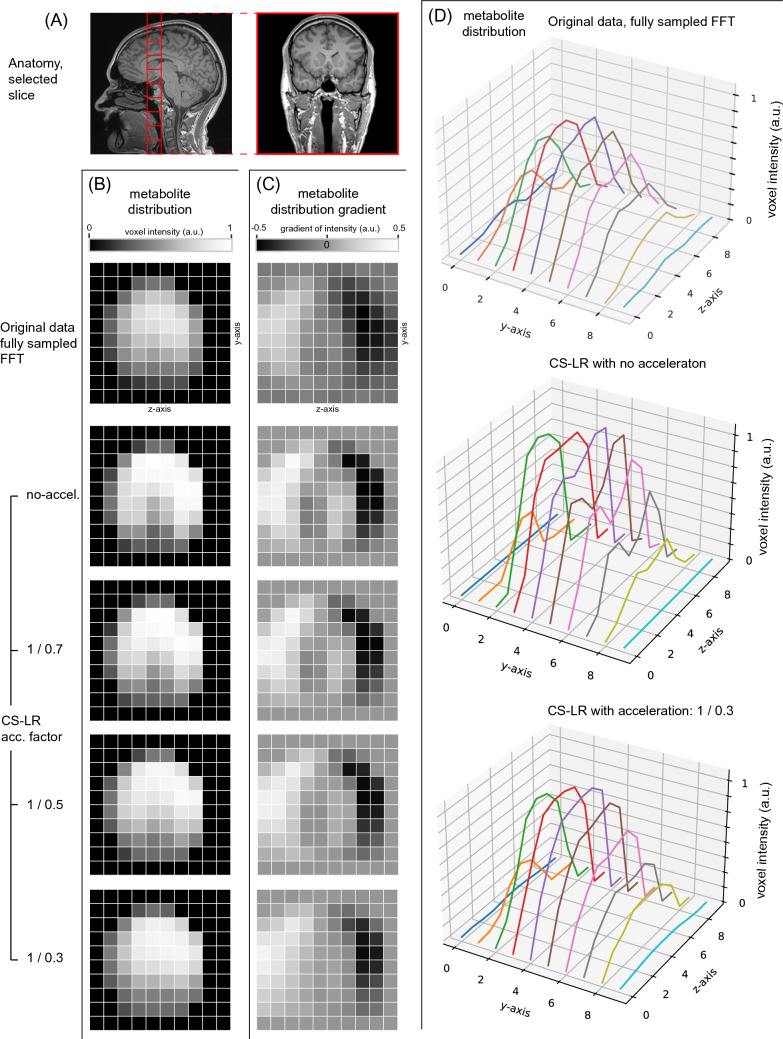
Effects of CS–LR method on metabolic mapping. **A** Displays the reference anatomical images in the coronal plane. **B** Maps of the α-ATP signal area with scaling are in arbitrary unit; all figures scaled to the overall maximum. Corresponding left-to-right gradient (**C**). The first row exhibits the FFT of the original data, while the succeeding rows display the reconstructed data using the CS–LR method. The second row illustrates the CS–LR method without acceleration, while the third, fourth, and fifth rows correspond to the CS–LR method with acceleration factors of 1.4, 2, and 3, respectively. **D** Depicts 3D plots of intensity profile corresponding to gradient maps of **panel C** with the FFT of the original data (top), the CS–LR reconstruction without acceleration (middle), and with acceleration of 3 (bottom)

Figure 4 displays the brain quantification mapping of *α*-ATP for the original data, and the CS–LR method, without and with acceleration factors of 1.42, 2, and 3. A coronal plane slice of subject 8 was arbitrarily chosen as illustrated by the anatomical reference images (Fig. 4A). Along with the metabolite quantification mapping (Fig. 4B), the gradient of the mapping is presented (Fig. 4C). The gradient was calculated from left to right and shows the intensity variation between voxels. For the original data, and the CS–LR method without and with an acceleration factor of 3, the metabolite maps are presented in 3D plots (Fig. 4D). The results present qualitatively an improvement in metabolite signal using CS–LR reconstruction, while maintaining spatial structures and edges during acceleration. This observation is further supported by the gradient maps in Fig. 4C, which exhibit sharper edge measurements of the CS–LR reconstruction in comparison with fully sampled FFT. The figure is intended to introduce and discuss the data used for the Sobel spatial evaluation method and the group analysis reported directly below.

Table 1 presents quantitative results demonstrating the CS–LR method's ability to enhance the complexity of acquired information. The table reports the combined three-axis edge magnitudes calculated using the Sobel kernel, which was applied to metabolite maps (PCr, ATPs, and Pi) from the brains of all ten subjects. The aggregated results are provided in the table. An example of a typical metabolite map used in this analysis is shown in Fig. 4B. The analysis of major metabolite maps (ATPs, PCr, and Pi) revealed significant improvements in edge detection with the implementation of CS–LR reconstruction compared to inverse FFT reconstruction, regardless of the acceleration factor used. Notably, 41 out of 45 conditions showed p values smaller than 10⁻^5^, with the remaining 4 conditions yielding p values smaller than 10⁻^4^.

**Table 1:** Edges magnitudes of brain metabolites distribution using Sobel kernel

	PCr	α-ATP	β-ATP	γ-ATP	Pi
IFFT	0.81 ± 0.05	0.85 ± 0.07	0.83 ± 0.06	0*.*86 ± 0*.*07	0*.*84 ± 0*.*05
LR no acc.	0.96 ± 0.09**	1.05 ± 0.09***	1.05 ± 0.07***	1.04 ± 0.07***	1.03 ± 0.09***
LR acc. 1.11	0.95 ± 0.11**	1.04 ± 0.09***	1.06 ± 0.07***	1.04 ± 0.08***	1.05 ± 0.08***
LR acc. 1.24	0.96 ± 0.12**	1.04 ± 0.09***	1.04 ± 0.08***	1.02 ± 0.09***	1.04 ± 0.09***
LR acc. 1.42	0.94 ± 0.12***	1.02 ± 0.08***	1.05 ± 0.07***	1.02 ± 0.08***	1.01 ± 0.10***
LR acc.1.66	0.94 ± 0.12***	1.02 ± 0.10***	1.04 ± 0.08***	1.01 ± 0.10***	1.02 ± 0.11***
LR acc. 2.00	0.97 ± 0.11***	1.03 ± 0.09***	1.04 ± 0.09***	1.03 ± 0.09***	1.07 ± 0.08***
LR acc. 2.50	0.96 ± 0.11***	1.02 ± 0.08***	1.03 ± 0.08***	1.02 ± 0.09**	1.04 ± 0.11***
LR acc.2.94	0.98 ± 0.10***	1.04 ± 0.10***	1.04 ± 0.08***	1.04 ± 0.09***	1.04 ± 0.08***
LR acc. 3.00	0.95 ± 0.11***	1.01 ± 0.10***	1.02 ± 0.10***	1.03 ± 0.10***	1.06 ± 0.12***

The table presents the RMS ± standard deviations of the edge magnitude of metabolite quantification map (PCr, ATPs, and Pi) using a Sobel kernel. Analysis of variance (ANOVA) was conducted to identify significant differences between the CS–LR method at each increasing acceleration factor compared to the FFT of the original data. Dunn–Šidák correction was applied to control the family-wise error rate, resulting in: *p ≤ 1 × 10^−3^, **p ≤ 1 × 10^−4^, and ***p ≤ 1 × 10^−5^

**Fig. 5:**
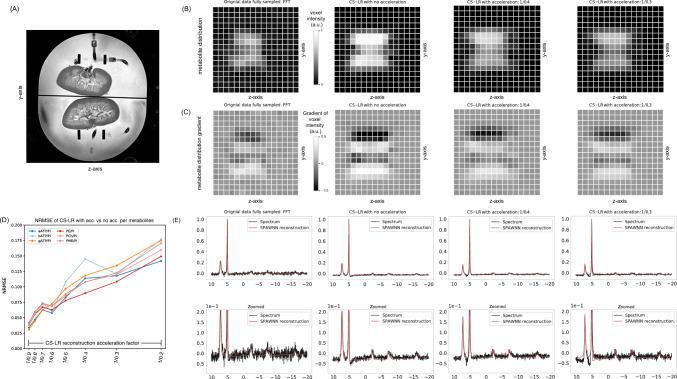
Application of the CS–LR acceleration and reconstruction methods on the kidney data: **A** displays the anatomical data in coronal plane and **B** shows the signal intensity maps of the *α*-ATP in the same plane with its corresponding gradient (**C**), which is computed with a down-to-up gradient. Voxel spectra from the corresponding mapping **E**: the first column corresponds to the FFT of the original data, the second column displays the CS–LR method without acceleration, and the 3rd and 4th columns show the CS–LR method with an acceleration of 2.5 and 3.33, respectively. The two downfield peaks are PME, resp, Pi (prominent peak, 25 mM used as reference for quantification). ATPs are better seen on the zoomed figures (E, bottom). NRMSE of the metabolic ratio estimation with respect to the Pi for the ATP and the PME (**D**)

Figure 5 presents analogous findings to those in the previous figure, but applied to kidney data to demonstrate the method's applicability to other organs. The figure showcases the anatomical slice (Fig. 5A) corresponding to the metabolite intensity maps of *α*-ATP (Fig. 5B), its corresponding gradient (Fig. 5C), and an example of voxel spectra for the FFT of the original data, the CS–LR method without and with acceleration factors of 2.5 and 3. The figure also presents NRMSE values for the metabolic quantification, with a reduced number of metabolites. Specifically, only ATPs, PME, and Pi are observed on the spectra, and the acceleration was extended to a maximum factor of 4. The analysis was also performed with the same 31P-SPAWNN model than the brain, highlighting its robustness to low SNR.

## Discussion

The current study examines the efficacy of the Low-Rank method in combination with Compressed Sensing (CS–LR) for acceleration and reconstruction of ^31^PMRSI. A unique feature of the presented acceleration is the use of different random patterns for each weighted average. The original data, reconstructed with a direct Fourier transform, were compared to those reconstructed using the CS–LR method both without acceleration and with varying acceleration factors. The use of acceleration has important practical implication, enabling significant acquisition-time savings, making ^31^P-MRSI more feasible in clinical settings. The results reported in this research are retrospective, obtained through a posteriori acceleration of ^31^P-MRSI data acquired from both brain and kidney tissues. One could argue that our acquisition scheme may disadvantage the standard CSI data using a 4-kHz receiver bandwidth, as a 2-kHz bandwidth would suffice to cover the useful 30 ppm range of ^31^P-MRS at 3 T. However, in our study, this setting is arbitrary and not critical, because the objective was to make a relative comparison under the same initial SNR conditions. Additionally, this study uses retrospective data acquired with a 4-kHz bandwidth, which was not considered critical at the time due to the use of apodization in the previous study. Moreover, the decision to forego apodization in the current study was driven by its tendency to cause peak broadening, leading to a loss of spectral resolution.

The acceleration was achieved by undersampling k-space while maintaining a 20 to 30% hard radius fully sampled at the center of k-space [39]. The technique leverages the elliptical weighted average to generate distinct random patterns for each average, consequently maintaining a higher k-space coverage. Overall, the acceleration results indicate that ^31^P-MRSI acquisition can be reduced to 12–15 min with an acceleration factor of 3 while keeping comparable data quality compared to no acceleration. SPAWNN was able to evaluate all spectra utilizing the same general training model. The LR methods used in this study indicated that *K* = 5 components were optimal for the specific application. Fewer components failed to provide enough information for accurate reconstruction, while more components introduced excessive noise, degrading the results. The regularization factor, *λ* = 1 × 10⁻^3^, was determined empirically. It was iteratively increased from an initial value of *λ* = 1 × 10⁻^5^. Values of *λ* below 1 × 10⁻^3^ led to reconstructions dominated by noise and insufficient signal, whereas values above this threshold resulted in over-regularization, which suppressed spatial details.

Our study demonstrates that the CS–LR method is as efficient as a matched filter method at improving the SNR compared to the original data and remains stable across acceleration factors. (Fig. 3A) Unlike other methods such as apodization and smoothing filters, which can cause peak broadening, the CS–LR method is preserving the original linewidth (Figs. 2, 3B, and S2).

The impact of the CS–LR methods on the accuracy of quantification is assessed with the NRMSE, as shown in Figs. 2 and 5. NRMSE calculations for concentration ratios revealed a linear relationship between the quantification error and the acceleration factor, and subsequently the undersampling. The metabolites with high signal and low spectral overlap, including the three resonances of ATP and Pi, exhibited an error of less than 15% in the ratio estimation with the removal of 2*/*3 of the k-space.

For acceleration factors below 2, the brain’s ATP, Pi, and PME over the PCr and kidney’s ATP, Pi, and PME over the Pi showed errors below 10%, indicating low deviation of the accelerated CS–LR data compared to the non-accelerated CS–LR data. However, for an acceleration of 3 in the brain and between 3.33 and 4 for the kidneys, the error estimation reached 15%. Metabolites with lower signal levels and greater spectral overlap, such as PDE and NAD, displayed NRMSE values as high as 30%. It is worth noting that the acceleration also induced errors, as previously reported in the literature [36, 37, 40]. Additionally, the effectiveness of the ^31^P-SPAWNN model in analyzing and reconstructing the MRSI data was demonstrated, as no adjustments were required to analyze the voxels spectra throughout the entire dataset.

The CS–LR method succeeded in recovering signal attenuation illustrated by the presence of the anatomical oral cavity, which is discernible up to an acceleration factor of 2 as illustrated in Fig. 4B. Moreover, the 3D plots in Fig. 4D emphasize the observation of signal attenuation at the center of the z-axis, with a clear reduction in signal intensity. As acceleration is applied, the structure becomes less evident, resulting in a smoothing effect and loss of spatial precision. Notably, the signal and gradient maps of the original data and their counterparts up to an acceleration factor of 3 display similar levels of information (Fig. 4C). Similarly, the images of the kidneys presented in Fig. 5 demonstrate the efficacy of the LR method in enhancing signal and edge sharpness. The loss of spatial information is a consequence of the loss of frequency information in the k-space domain, which leads to a smoothing effect, as depicted in Figs. 4 and 5. Since the reconstruction is non-linear, the loss of spatial information cannot be precisely quantified or recovered [39, 41]. In addition to the edge preservation analysis achieved on metabolite quantification maps presented above, we conducted a Root-Mean-Square Radius (RMSR) analysis of the point spread function (PSF) to further validate the spatial localization capabilities of the CS–LR method. This analysis compares our acceleration scheme to a fixed undersampling mask applied across all averages (see supplementary material S1). It is important to note that this PSF analysis relies on the direct Fourier transform of the sampling pattern and does not account for the advantages of solving the inverse problem or the regularization incorporated in the CS–LR framework.

Table 1 displays the quantitative evaluation of edge detection using the FFT of the original data and the CS–LR methods. The CS–LR methods without acceleration showed a significant improvement in edge sharpness for all metabolites and acceleration factors compared to FFT. An increase in standard deviation was observed with increasing acceleration factor, indicating a decrease in measurement precision, consistent with the less precise measures seen in the NRMSE for the concentration in Fig. 2.

The analysis of kidney data in Fig. 5 yielded results similar to those observed in brain data analysis. Application of the CS–LR method resulted in improved SNR, particularly for the low signal of ATP. The *α*-ATP intensity map exhibited sharper signal resolution with clear contrast on the gradient map. For the kidney data, an acceleration factor of 3 resulted in higher signal resolution and a sharper gradient image than the original data. Similar to the brain data, the NRMSE exhibited a linear increase in error with acceleration, with an error of less than 20% for measured metabolites at an acceleration factor of 4 and less than 15% for an acceleration of 3.

The enhancement in SNR while keeping the linewidth constant for all metabolite resonance peaks provides distinct benefits for more accurate analysis on the image reconstruction and sharpness. With acceleration, the CS–LR method provides the potential to acquire data at a faster rate, albeit with an inherent trade-off in spatial resolution and errors in metabolite quantification. Our results demonstrate that up to an acceleration of 2 for brain sequence acquisition and 3 for excised kidney sequence acquisition, the spatial loss and quantification errors remain within a 10% range. The ability to accelerate acquisition is highly desirable in clinical settings to ensure patient comfort and avoid lengthy acquisitions that could degrade data quality. This is particularly relevant in the context of the excised kidney scan, as the measurement of metabolite concentration in organs is crucial for ongoing research evaluating their viability for transplantation. Thus, a fast acquisition is a critical requirement in such applications.

A potential aspect of the CS–LR method that has yet to be explored in this study is the potential to utilize the acceleration to achieve an acquisition with the same acquisition time but at a higher resolution. In our ongoing research, we intend to modify the FID-MRSI sequence to include the acceleration into the acquisition protocol. One limitation of our experiment was the lack of multi-channel capability in the ^31^P-MRSI coil. Incorporating multi-channel measurements from multiple spatial points, coupled with the coil sensitivity profile, would have provided more information for reconstruction and possibly improved results.

In a recent study by Santos-Díaz et al. [62], CS was combined with echo-planar spectroscopic imaging (EPSI) to accelerate the acquisition of dynamic ^31^P-MRSI data. The researchers successfully applied CS to accelerate the acquisition of an 8 × 8 matrix by a factor of 2.7. In another study by Santos-Díaz et al. [63], the authors compared the performance of the CS model and the LR model, both in combination with a flyback EPSI sequence. Their study demonstrated that the spectral quality was well preserved at accelerations of 2 and 3 using two CS reconstruction methods (one using L1 norm minimization and the other using low-rank Hankel matrix completion). Their study demonstrated that the LR approach for reconstruction outperformed the CS methods at up to threefold acceleration with lower NMRSE value at all acceleration factor. This is in line with our results using combined CS–LR reconstruction.

In a study conducted by Tavakkoli et al. [64], optimization of CS random pattern was investigated. Such optimization was not performed due to the limited k-space coverage but could be envisaged in a future work especially if the k-space coverage is extended.

In summary, we have presented a novel acquisition/reconstruction scheme, CS–LR, for the FID-MRSI sequence. A distinguishing aspect of this CS–LR method is the implementation of distinct random undersampling patterns for each weighted average. This approach of random k-space undersampling accelerates scan time and enables faster acquisition. Additionally, the reconstruction method has demonstrated to be efficient in preserving the spectral and spatial quality of the data at 3 T.

## Supplementary Information

Below is the link to the electronic supplementary material.

Supplementary file1 (PDF 951 KB)

## Data Availability

The datasets used during the current study are available upon reasonable request at 10.26037/yareta:udek3gfdwjfvhbmo5ohlhzfd4i (Lazeyras, F. Project 182658 Kidney Results, 2023) and 10.26037/yareta:kmtgtfx3ezgwbeoowozwipzq5a (Courvoisier, S. 31P MRSI acquisitions, 10 brains from healthy subjects, 2023).
