# Supplementary material for: Improved phosphorus MRSI acquisition through compressed sensing acceleration combined with low-rank reconstruction
Source: MAGMA. 2024 Dec 27;38(2):161–73. doi: 10.1007/s10334-024-01218-y (PMC11914371; doi:10.1007/s10334-024-01218-y)
Supplement: Supplementary file 1 — Supplementary file1 (PDF 951 KB) [file 10334_2024_1218_MOESM1_ESM.pdf]

Supplementary material for article ”Improved Phosphorus MRSI acquisition through Compressed Sensing Acceleration combined with Low-Rank Reconstruction”

Songeon, Julien Lazeyras, François Agius, Thomas
Dabrowski, Oscar Ruttiman, Raphael Toso, Christian
Longchamp, Alban Klauser, Antoine
Courvoisier, Sebastien

December 9, 2024

associated with article:

DOI: 10.1007/s10334-024-01218-y

S1 Point spread function energy dispersion evaluation

To effectively characterize the overall extend of the point spread function (PSF) associated with a sampling strategy, we estimated the global energy dispersion of the PSF center by computing the Root Mean Square Radius (RMSR) metric on the Fourier transform of the integral of the k-space acquisition masks of averages (1 where acquired, 0 otherwise). The RMSR is defined as :

$$\mathrm{RMSR}(\boldsymbol{P}) = \sqrt{\mathbb{E}[\mathrm{vec}((\boldsymbol{D} \odot \boldsymbol{P})^{②})]} \tag{1}$$

where $\boldsymbol{P} \in \mathbb{R}^{m \times n}$ is the PSF magnitude image, $\boldsymbol{D} \in \mathbb{R}^{m \times n}$ contains Euclidean distances to the center of $\boldsymbol{P}$, vec is the vectorization operator and we define the symbol ② as the Hadamard (point-wise) square. The following three acquisition scenarios were considered:

1. 24-averages masks used by the default by the pulse sequence,
2. Independent pseudo-random mask at each average,
3. One fixed pseudo-random mask for all averages.

Figure S1.1 displays examples of sampling densities and their corresponding PSF obtained by Fourier transform. The k-spaces acquisition densities shown are the central k-space plane with acceleration factor of 2 for both the independent pseudo-random sampling (scenario 2, black line) and fixed one (scenario 3, blue line). The corresponding PSF obtained by Fourier transform of the k-space density are shown underneath. The PSF RMSR values for the three scenarios defined above are reported in **Figure S1.2** averaging the results of 10 generated examples per acceleration factor. The much smaller overall RMSR show a clear advantage in term of spatial localization for the independent pseudo-random mask at each average compared to a fixed pseudo-random one over all averages. For the third scenario, the steep increase of RMSR (even for low acceleration) is due to strong aliasing produced by the missing k-space points across all averages. For the second scenario, the RMSR slope steepness is much smaller, thanks to independent pseudo-random masks preserving the overall (summing all averages) sampling distribution shape and avoiding occurrence of the totally missing k-space points. Some missing point likely start to appear at acceleration factor 1.4 (1/0.7) where we can see an elbow on the curve. Also above an acceleration factor of 2.5 we see a reduction of RMSR that is not related to an improvement of localization but an increase of blurring.

RMSR gives us only an partial insight over the acquisition acceleration scheme through PSF energy localization. In our case, it is limited to the direct Fourier transform and it does not take into account the advantages of solving the inverse problem and the regularization embedded into the CS-LR framework.

S2 Additional Pi, ATP and PME peak width results for figure 3C

Figure S2.1 displays peak width measurements of Pi, ATP, and PME similarly to the linewidth PCr results presented in Figure 3C of the main article.

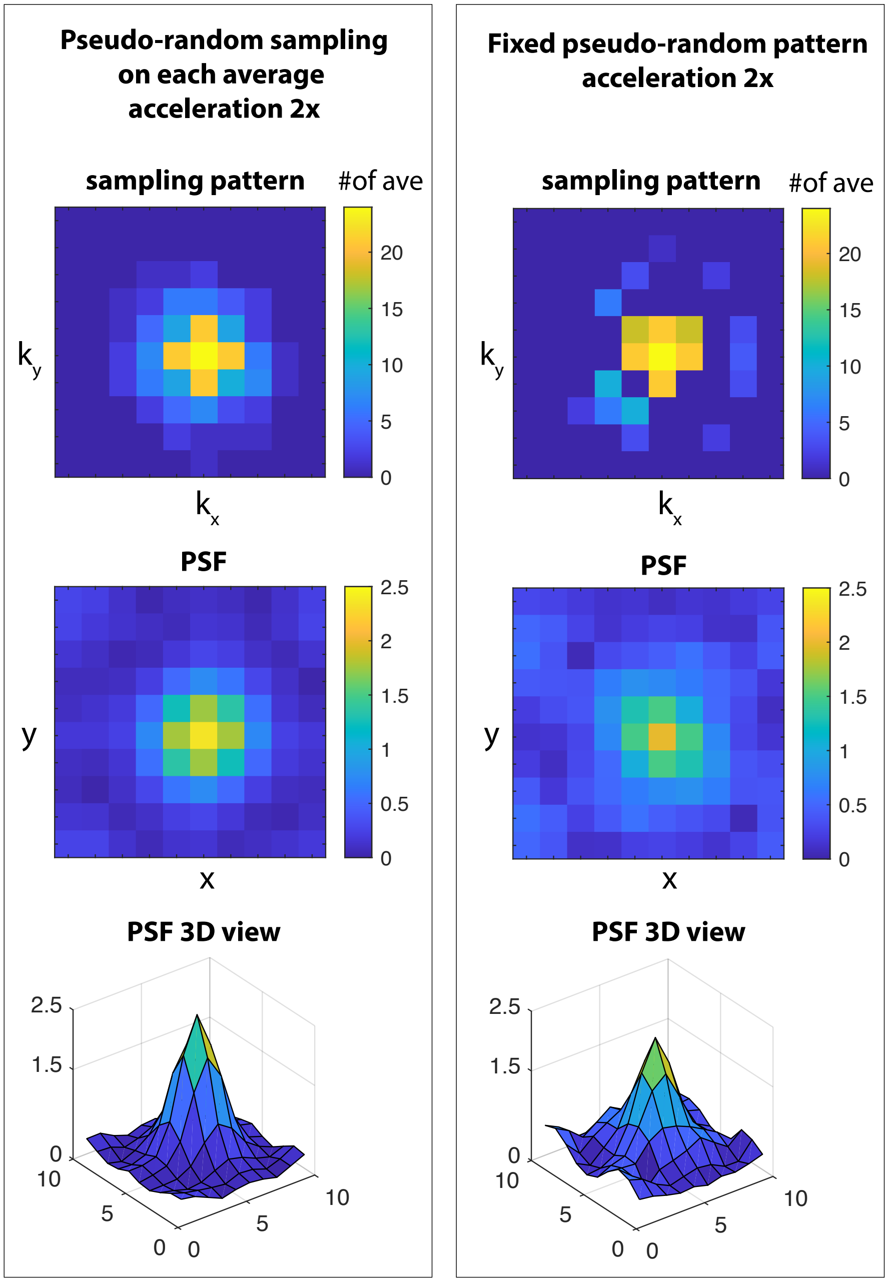

Figure S1.1: k-space sampling densities for the central k-space plane using an acceleration factor of 2 (top) for both the independent pseudo-random sampling (left) and fixed pseudo-random pattern one (right); corresponding PSF for both strategies (center) and 3D view of the PSF to better see the aliasing and central peak(bottom).

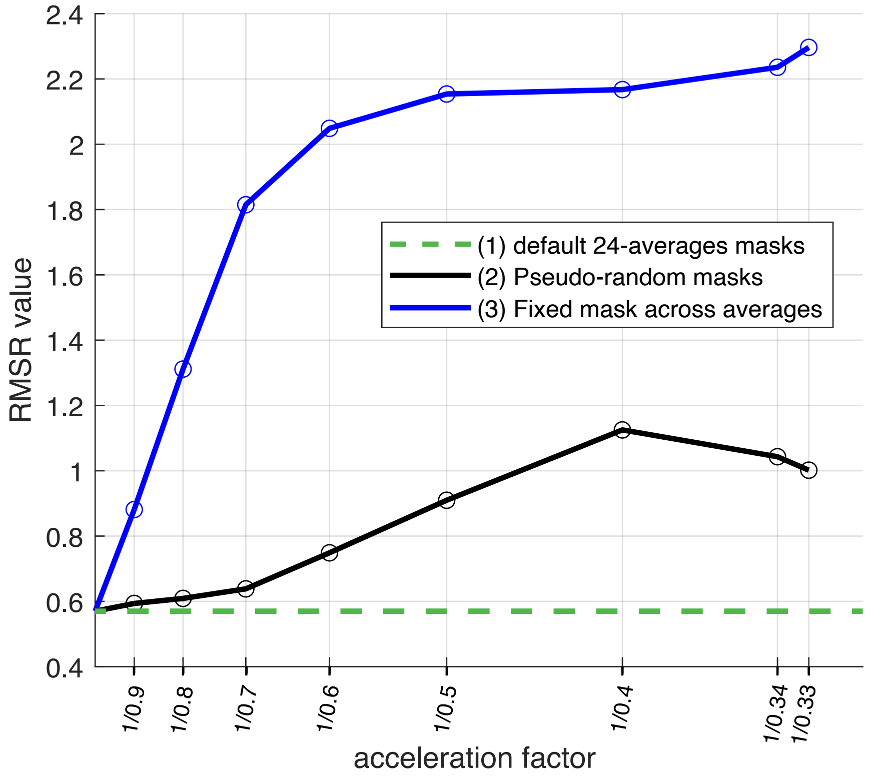

Figure S1.2: RMSR values computed on the PSF of the three k-space sampling scenarios. For scenarios 2 and 3, 10 samples were generated for each acceleration factor.

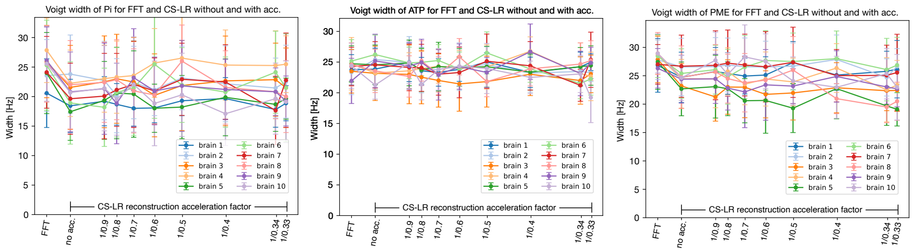

Figure S2.1: This supplementary figure presents the peak width measurements of Pi, ATP, and PME. These peak width measurements were obtained using the same data (^{31}P-MRSI acquisitions of 10 healthy subject brain) and experimental setup as Figure 3.C, with the FFT of the original data and the CS-LR reconstruction with increasing acceleration factor. These measurements provide additional insights into the impact of the reconstruction methods on the spectral peaks.
